# Association between cardiac autonomic function and physical activity in patients at high risk of sudden cardiac death: a cohort study

**DOI:** 10.1186/s12966-021-01200-0

**Published:** 2021-09-20

**Authors:** Xuerong Sun, Shuang Zhao, Keping Chen, Wei Hua, Yangang Su, Xin Liu, Wei Xu, Fang Wang, Xiaohan Fan, Yan Dai, Zhimin Liu, Shu Zhang

**Affiliations:** 1 Arrhythmia Center, State Key Laboratory of Cardiovascular Disease, Fuwai Hospital, National Center for Cardiovascular Diseases, Chinese Academy of Medical Sciences and Peking Union Medical College, 167 Bei Li Shi Road, Xicheng District, Beijing, 100037 China; 2 Department of Cardiology, Shanghai Institute of Cardiovascular Diseases, Zhongshan Hospital, Fudan University, Shanghai, China; 3 Department of Cardiology, Beijing Anzhen Hospital, Capital Medical University, Beijing, China; 4 Department of Cardiology, Nanjing Drum Tower Hospital, Nanjing, China; 5 Department of Cardiology, Shanghai First People’s Hospital, Shanghai Jiao Tong University School of Medicine, Shanghai, China

**Keywords:** Physical activity, Heart rate variability, Implantable cardioverter defibrillator, Cardiac death, All-cause mortality, Causal mediation analysis

## Abstract

**Background:**

High levels of physical activity (PA) and heart rate variability (HRV) are associated with cardiovascular benefits in patients with cardiovascular diseases. HRV, representing cardiac autonomic function, is positively associated with PA. However, the impacts of PA and cardiac autonomic function on cardiovascular outcomes were not analysed in the same study population. This lack of evidence supported our hypothesis that PA might contribute to cardiovascular benefits via enhanced cardiac autonomic function.

**Methods:**

Patients with implantable cardioverter defibrillator (ICD) or cardiac resynchronisation therapy defibrillator (CRT-D) implantation were included from the SUMMIT registry. HRV and PA values were assessed during the first 30–60 days post device implantation using a continuous home monitoring system. Causal mediation analysis was conducted to explore the possible mediation function of HRV in the association of PA with long-term cardiac death and all-cause mortality in patients at a high risk of sudden cardiac death.

**Results:**

Over a mean follow-up period of 47.7 months, 63 cardiac deaths (18.9%) and 85 all-cause death events (25.5%) were observed among 342 patients with ICD/CRT-D implantation. A positive linear association between HRV and PA was demonstrated and the β value of HRV was 0.842 (95% confidence interval [CI]: 0.261–1.425, *P* = 0.005) in the multiple linear regression analysis. Multivariable Cox proportional hazards analysis revealed that high levels of PA (≥11.0%) and HRV (≥75.9 ms) were independent protective factors against cardiac death (PA: hazard ratio [HR] = 0.273; 95% CI, 0.142–0.526, *P* < 0.001; HRV: HR = 0.224; 95% CI, 0.103–0.489, P < 0.001) and all-cause mortality (PA: HR = 0.299; 95% CI, 0.177–0.505, P < 0.001; HRV: HR = 0.394; 95% CI, 0.231–0.674, *P* = 0.001). Causal mediation analysis demonstrated partial mediation effects of PA that were mediated through HRV on cardiac death (mediation proportion = 12.9, 95%CI: 2.2–32.0%, *P* = 0.006) and all-cause mortality (mediation proportion = 8.2, 95%CI: 1.6–20.0%, P = 0.006).

**Conclusions:**

HRV might be a modest mediator in the association between high levels of PA and the reduced risks of cardiac death and all-cause mortality in ICD/CRT-D recipients. This finding supports that enhanced cardiac autonomic function might be one of the underlying mechanisms by which regular PA contributes to cardiovascular benefits.

## Introduction

Physical activity (PA) refers to any bodily movement resulting from skeletal muscle action, which can reflect the status of energy expenditure or exercise intensity of an individual [1, 2]. Previous studies have reported that high levels of PA can protect against cardiovascular adverse events, cardiovascular death, and all-cause mortality in individuals with or without cardiovascular diseases (CVDs) [3–5]. Moreover, the dose-response relationships between the extent of PA and the risks of cardiac death and all-cause mortality were assessed to determine the optimal beneficial intervals [3, 6, 7]. Consequently, appropriate exercise-based cardiac rehabilitation or exercise training was recommended for patients with coronary heart diseases or chronic heart failure (HF) [3]. However, the potential underlying mechanism through which regular PA contributed to cardiovascular benefits remains unclear.

PA was previously assessed using self-reported structured questionnaires; however, this assessment had certain biases and errors depending on the individuals’ educational level and cognitive function [8, 9]. In recent studies, the values of the objective accelerometer-measured PA have been widely recognised [10]. Objective accelerometer-derived PA has a positive association with heart rate variability (HRV) [11–13]. HRV is a non-invasive and easily measured parameter in a time-dominant or frequency-dominant form and reflects cardiac autonomic function [14, 15]. Dysfunction of the autonomic nervous system also plays a crucial role in the clinical course of CVDs, and low levels of HRV indices are associated with increased risks of cardiovascular adverse events and mortality [16, 17]. Moreover, available evidence implies that regular PA might protect against death partly by enhanced cardiac autonomic function [11]. Nevertheless, a lack of studies evaluates the associations among PA, HRV, and long-term mortality in the same study population. The possible mediation function of cardiac autonomic function in the association between PA and long-term mortality demands further discussion.

Cardiac implantable electronic devices, such as implantable cardioverter defibrillator (ICD) or cardiac resynchronisation therapy defibrillator (CRT-D), can provide daily PA and HRV data that can be routinely collected using a continuous remote home monitoring system [18]. Therefore, in the present study, we performed a causal mediation analysis of the data from ICD/CRT-D recipients and aimed to 1) explore the causal chains among PA, HRV, and long-term mortality, 2) evaluate the possible mediation function of HRV in the association between high levels of PA and reduced risks of cardiac death and all-cause mortality, and 3) derive some evidence supporting that PA might contribute to cardiovascular benefits via enhanced cardiac autonomic function.

## Methods

### Study participants

In the present study, we conducted a retrospective analysis using the archived home monitoring transmission data from the Study of Home Monitoring System Safety and Efficacy in Cardiac Implantable Electronic Device-implanted Patients (SUMMIT) registry [7]. Patients who underwent ICD or CRT-D implantation between May 2010 and April 2014 were included upon meeting the following criteria: 1) ICD or CRT-D implantation in accordance with the recommendations outlined in the current guideline [19]; 2) the equipment with a remote home monitoring system and initiation of continuous monitoring after implantation; 3) capacity to supply available data related to daily HRV and PA; 4) age of the patients ≥18 years at implantation; and 5) life expectancy > 1 year after device implantation. Exclusion criteria were as follows: 1) diagnosis of persistent, long-standing, or permanent atrial fibrillation (AF); 2) AF episodes occurring during the first 30–60 days after implantation; 3) > 5% of atrial or ventricular pacing percentages during the window period of HRV measurement; 4) missing or incomplete home monitoring data; 5) loss to follow-up; 6) diagnosis of a malignant tumour; or 7) schedule for heart transplantation.

This study complied with the Declaration of Helsinki and was approved by the ethics committee of Fuwai Hospital (the chief institute) and all other participating organisations. All patients provided written informed consent before study participation.

### Collection of baseline characteristics

Data regarding baseline characteristics, including demographic characteristics (sex, age at implantation, body mass index [BMI], ICD or CRT-D implantation, and New York Heart Association [NYHA] Class III-IV), echocardiographic characteristics (left ventricular ejection fraction [LVEF], left ventricular end-diastolic dimension [LVEDD]), comorbidities (hypertension, diabetic mellitus [DM], stroke, dilated cardiomyopathy [DCM], hypertrophic cardiomyopathy [HCM], ischaemic cardiomyopathy [ICM], valve disease, prior myocardial infarction [MI], percutaneous coronary intervention [PCI], coronary artery bypass grafting [CABG], prior paroxysmal AF, long QT syndrome [LQTS], and pre-implant syncope), and medication (angiotensin-converting enzyme inhibitors or angiotensin receptor blockers [ACEIs/ARBs], aldosterone antagonists, diuretics, statins, calcium channel blockers [CCBs], beta-blockers, amiodarone, and antiplatelets), were collected from medical records.

### Measurement of PA and HRV

Daily PA and HRV were detected using ICD devices (Biotronik, Germany). HRV and PA at baseline were assessed during the first 30–60 days after the ICD/CRT-D implantation, in accordance with the recommendations of previous studies [20]. The average values of daily PA and HRV at baseline were calculated for each patient.

PA was measured using the devices’ acceleration sensors. PA was expressed as a percentage of the active time per day when the recorded rates were higher than basic rates, where 20% indicated 4.8 h of daily PA, with a resolution of 2 s. The accuracy of these acceleration sensors has been validated using the treadmill exercise [21].

HRV was measured in a time domain analysis and quantified as the standard deviation (SD) of the normal P-P intervals (SDNN algorithm) over a 24-h period [11, 15]. High levels of HRV can represent better joint sympathetic and parasympathetic modulation of heart rate [14, 15]. To obtain the reliable results of HRV analysis, the average values of daily atrial and ventricular pacing percentages should be both ≤5% during the window period of HRV measurement [22, 23].

### Follow-up and clinical outcomes

Data regarding PA and HRV were recorded and transmitted using a remote home monitoring system every day. If data transmission was disrupted, the clinical research coordinator contacted the patients or their family members and confirmed the patients’ health conditions immediately. Regular telephonic interviews or clinic visits were conducted to collect the information about clinical outcomes during follow-up. Data regarding the date of death and cause of death were obtained from the death certificate provided by family members. The primary endpoint was cardiac death (ICD-10: I00 to I09, I11, I20 to I51), and the secondary endpoint was all-cause mortality.

### Grouping

Based on the baseline PA tertiles, patients were categorized into three groups: PA tertile 1 (range, 1.1–7.8%; *n* = 114), PA tertile 2 (range, 7.8–13.0%; n = 114), and PA tertile 3 (range, 13.0–33.3%; n = 114).

### Statistical analysis

The main exposure variables included PA and HRV. Continuous variables are presented as means ± SDs, and categorical variables are presented as numbers and percentages. Baseline characteristics were compared between groups using one-way analyses of variance for continuous variables and the chi-squared tests for categorical variables. Rates of cardiac death and all-cause mortality events were calculated, and the difference was compared using the chi-squared test.

**Fig. 1:**
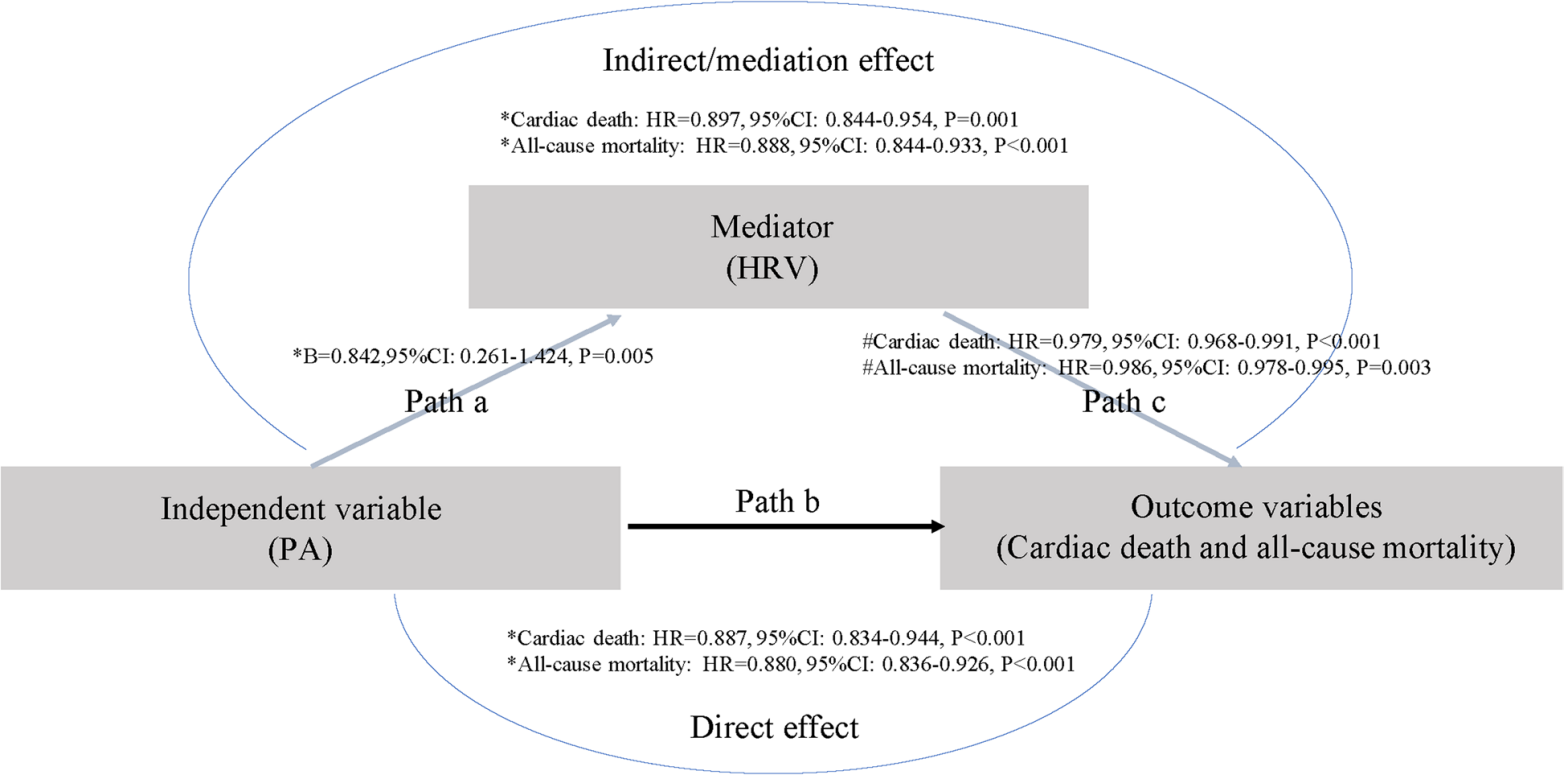
Path diagram of the causal mediation model with a three-variable system. In Path a, PA (independent variable) has a significantly positive relationship with HRV (mediator) in a multiple linear regression model. In Path b, PA (independent variable) was shown as an independent predictor for long-term cardiac death and all-cause mortality (outcome variables) in the multivariable Cox regression model without HRV. In Path c, both PA (independent variable) and HRV (mediator) remained significant to predict the outcome variables using the multivariable Cox regression model. Thus, this mediation analysis confirmed the partial mediation function of HRV. CI: confidence interval; HRV: heart rate variability; HR: hazard ratio; PA: physical activity. * each additional 1% increase in PA. # each additional 1 ms increase in HRV

A box plot was generated to display the distribution of HRV based on different PA levels at baseline, and a scatter plot was used to describe the association between PA and HRV. Simple and multiple linear regression models were used to further explore their linear relations (Fig. 1, Path a). Multiple linear regression analysis considered the impacts from potential confounding variables, including age at implantation, sex, BMI, LVEF, LVEDD, ICD or CRT-D implantation, NYHA Class, hypertension, DM, stroke, DCM, ICM, MI, PCI, pre-implant syncope, prior AF, uses of ACEIs/ARBs, diuretics, and aldosterone antagonists, which should maintain consistent with those in the multivariable Cox regression models.

Restricted cubic splines were used to flexibly model and visualize the associations between PA or HRV and hazard ratios (HRs) for long-term clinical outcomes, as well as to explore their cut-off values for cardiac death and all-cause mortality based on the inflection points demonstrated by the smooth curve fitting. Univariable and multivariable Cox proportional hazards models were used to evaluate the independent predictive values of PA and HRV for long-term cardiac death and all-cause mortality (Fig. 1, Path b and c). Either PA or HRV entered the univariable Cox regression models. Variables selected for in the multivariable analysis were those with a *P* value of < 0.05 in the univariable models and other potential confounders. Multivariable Cox regression model 1 was adjusted for age at implantation, sex, BMI, LVEF, LVEDD, ICD or CRT-D implantation, NYHA Class, hypertension, DM, stroke, DCM, ICM, MI, PCI, pre-implant syncope, prior AF, uses of ACEIs/ARBs, diuretics, and aldosterone antagonists. Multivariable Cox regression model 2 was adjusted for the above-mentioned confounders and additional HRV or PA. HRs and 95% confidence intervals (CIs) were calculated to show the impact at each level. A *P* value < 0.05 was considered significant, and all tests were two-sided.

Causal mediation analysis was conducted to assess the possible mediation function of HRV in the association between PA and long-term mortality. The mediation function represented the extent of PA that was able to influence the long-term mortality through modulating cardiac autonomic function. A causal mediation model with a three-variable system was established following the Baron and Kenny’s procedure [24, 25]. A path diagram is used to depict the causal chains (i.e., Path a-c) among PA (independent variable), HRV (mediator), and cardiac death and all-cause mortality (outcome variables) (Fig. 1). The mediation function is present when the following three conditions are met: a) PA is significantly associated with HRV in the multiple linear regression model (i.e., Path a, variations in the independent variable significantly accounted for the variations in presumed mediator); b) PA is shown as an independent predictor for cardiac death and all-cause mortality in the multivariable Cox regression model without HRV (i.e., Path b, variations in the independent variable significantly account for the outcome variables); c) HRV can independently predict the risks of cardiac death and all-cause mortality using the multivariable Cox regression model in which PA and HRV are simultaneously included (i.e., Path c, variations in presumed mediator significantly account for the outcome variables).

In Path c, if the effect of PA on clinical outcomes completely disappears, HRV fully mediates the association between PA and outcomes (full mediation), indicating that HRV as a single, dominant mediator; if the effect of PA still exits, HRV partially mediates the association between PA and outcomes (partial mediation), indicating the operation of multiple mediating factors. In addition, the R mediation package was used to estimate the average causal mediation effect (ACME) and the mediation proportion (prop. mediated) of PA on long-term mortality mediated through HRV [26]. The quasi-Bayesian Monte Carlo method was used to calculate the point estimates, 95% CI values and *P* values [27].

Statistical analyses were conducted using SPSS Statistics version 23.0 (IBM Corp., Armonk, NY) and R version 4.0.3 (Bunny-Wunnies Freak Out, The R Foundation for Statistical Computing, Vienna, Austria).

## Results

### Baseline characteristics

**Fig. 2:**
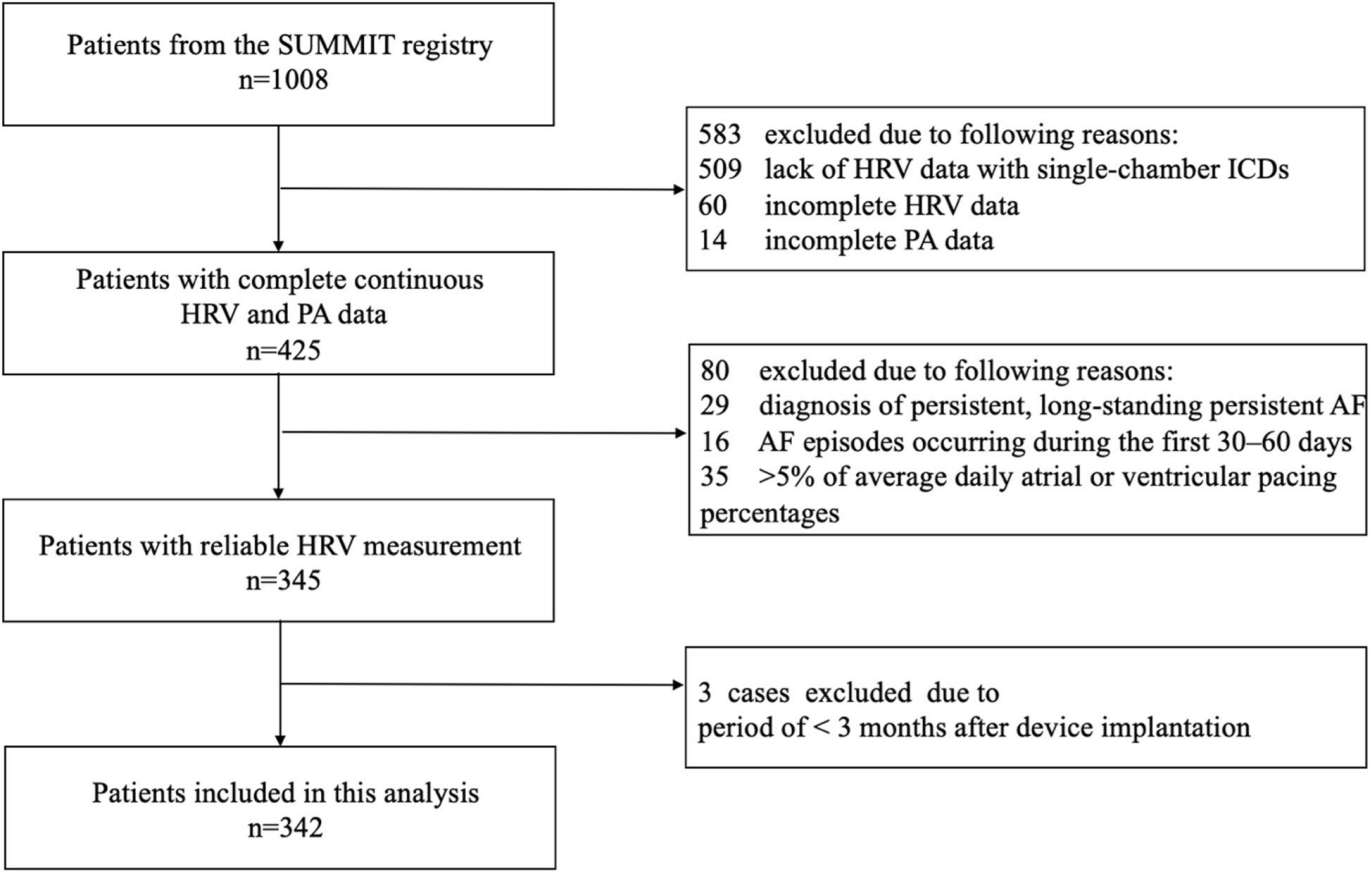
Flow chart for patient selection. AF: atrial fibrillation; HRV: heart rate variability; ICD: implantable cardioverter-defibrillator; PA: physical activity

In this retrospective analysis, 342 out of 1008 patients with HRV and PA data were included. Patients were excluded due to lack of continuous HRV data availability with single-chamber ICDs (*n* = 509), incomplete HRV data (*n* = 60), incomplete PA data (*n* = 14), diagnosis of persistent, long-standing persistent AF, AF episodes occurring during the first 30–60 days (*n* = 45), and > 5% of average daily atrial or ventricular pacing percentages (*n* = 35), or period of < 3 months after device implantation (n = 3). Figure 2 shows the flow chart for patient selection.

The mean age at implantation was 62.5 ± 13.4 years, and the male sex was dominant in the study cohort (78.1%). CRT-Ds were implanted in 184 (53.8%) patients and the mean LVEF was 39.13 ± 14.43%. The mean PA and HRV were 10.70 ± 5.46% and 72.52 ± 27.07 ms, respectively. Significant differences in PA (*P* < 0.001), HRV (P < 0.001), age at implantation (P < 0.001), hypertension (*P* = 0.003), stroke (*P* = 0.001), ICM (*P* = 0.021), PCI (P < 0.001) and CCBs usage (*P* = 0.019) were observed among different levels of baseline PA. Table 1 illustrates the comparison of baseline characteristics across three groups with different baseline PA levels.

**Table 1:** Baseline clinical characteristics

Parameters	Total (***n*** = 342)	Tertile 1 (n = 114)	Tertile 2 (n = 114)	Tertile 3 (n = 114)	P value
**Home monitoring data**					
Baseline PA, %	10.80 ± 5.35	5.18 ± 2.01	10.30 ± 1.48	16.93 ± 3.19	–
Baseline HRV, ms	71.14 ± 27.63	62.40 ± 25.43	72.23 ± 26.87	78.80 ± 28.85	–
**Demographics**					
Gender, male	78.4% (268)	76.3% (87)	74.6% (85)	84.2% (96)	*P* = 0.169
Age at implantation, years	62.37 ± 13.48	66.32 ± 13.66	63.26 ± 13.07	57.52 ± 12.26	*P* < 0.001
BMI, Kg/m^2^	23.56 ± 2.87	23.62 ± 2.85	23.53 ± 3.26	23.72 ± 2.45	*P* = 0.890
CRT-D implantation	53.8% (184)	51.8% (59)	57.9% (66)	51.8% (59)	*P* = 0.562
NYHA, Class III-IV	61.4% (210)	67.5% (77)	61.4% (70)	55.3% (63)	*P* = 0.163
**Echocardiography**					
LVEF, %	39.13 ± 14.43	38.64 ± 14.61	39.48 ± 14.20	39.29 ± 14.60	*P* = 0.900
LVEDD, mm	62.38 ± 13.20	60.17 ± 11.36	62.76 ± 13.69	64.23 ± 14.18	*P* = 0.066
**Comorbidities**					
Hypertension	35.1% (120)	39.5% (45)	43.0% (49)	22.8% (26)	P = 0.003
DM	14.6% (50)	17.5% (20)	14.0% (16)	12.3% (14)	*P* = 0.519
Stroke	2.6% (9)	7.0% (8)	0.9% (1)	0.0% (0)	P = 0.001
DCM	29.8% (102)	28.9% (33)	29.8% (34)	30.7% (35)	*P* = 0.959
HCM	3.8% (13)	0.9% (1)	5.3% (6)	5.3% (6)	*P* = 0.081
ICM	39.8% (136)	47.7% (54)	42.1% (48)	29.8% (34)	P = 0.021
Valve disease	2.3% (8)	3.5% (4)	1.8% (2)	1.8% (2)	*P* = 0.671
Prior MI	14.0% (48)	20.2% (23)	12.3% (14)	9.6% (11)	*P* = 0.059
PCI	11.7% (40)	21.1% (24)	9.6% (11)	4.4% (5)	P < 0.001
CABG	1.8% (6)	3.5% (4)	0.0% (0)	1.8% (2)	*P* = 0.060
Prior paroxysmal AF	7.6% (26)	9.6% (11)	7.0% (8)	6.1% (7)	*P* = 0.582
LQTS	1.5% (5)	0.9% (1)	1.8% (2)	1.8% (2)	*P* = 0.801
Pre-implant syncope	15.8% (54)	19.3% (22)	9.6% (11)	18.4% (21)	*P* = 0.087
**Medication**					
ACEIs/ARBs	41.5% (142)	43.0% (49)	43.9% (50)	37.7% (43)	*P* = 0.596
Diuretics	33.0% (113)	39.5% (45)	31.6% (36)	28.1% (32)	*P* = 0.172
Aldosterone antagonists	45.0% (154)	51.8% (59)	41.2% (47)	42.1% (48)	*P* = 0.208
CCBs	10.2% (35)	2.0% (7)	5.6% (19)	2.6% (9)	P = 0.019
Statins	22.8% (78)	24.6% (28)	28.1% (32)	15.8% (18)	*P* = 0.075
Beta-blockers	57.6% (197)	57.0% (65)	57.9% (66)	57.9% (66)	*P* = 0.988
Amiodarone	28.1% (96)	27.2% (31)	23.7% (27)	33.3% (38)	*P* = 0.260
Antiplatelets	21.6% (74)	27.2% (31)	22.8% (26)	14.9% (17)	*P* = 0.074

*ACEIs* angiotensin-converting enzyme inhibitors, *AF* atrial fibrillation, *ARBs* angiotensin receptor blockers, *BMI* body mass index, *CABG* coronary artery bypass grafting, *CCB* calcium channel blockers, *CRT-D* cardiac resynchronization therapy with defibrillation, *DCM* dilated cardiomyopathy, *DM* diabetes mellitus, *HCM* hypertrophic cardiomyopathy, *HRV* heart rate variability, *ICM* ischemic cardiomyopathy, *LQTS* long QT syndrome, *LVEF* left ventricular ejection fraction, *LVEDD* left ventricular end-diastolic dimension, *MI* myocardial infarction, *PA* physical activity, *PCI* percutaneous coronary intervention

### Clinical outcomes

Over a mean follow-up duration of 47.7 ± 20.8 months, 62 cardiac deaths (18.1%) and 94 all-cause mortality events (27.5%) were observed. As baseline PA increased from tertile 1 to tertile 2 to tertile 3 (5.18 ± 2.01% vs. 10.30 ± 1.48% vs. 16.93 ± 3.19%), the corresponding HRV increased continuously (62.40 ± 25.43 ms vs. 72.23 ± 26.87 ms vs. 78.90 ± 28.25 ms), but the incidence rates of cardiac death (26.3% vs. 18.4% vs. 9.6%, *P* = 0.005) and all-cause mortality (42.1% vs. 29.8% vs. 10.5%, *P* = 0.001) decreased continuously.

### Relationship between PA and HRV

**Fig. 3:**
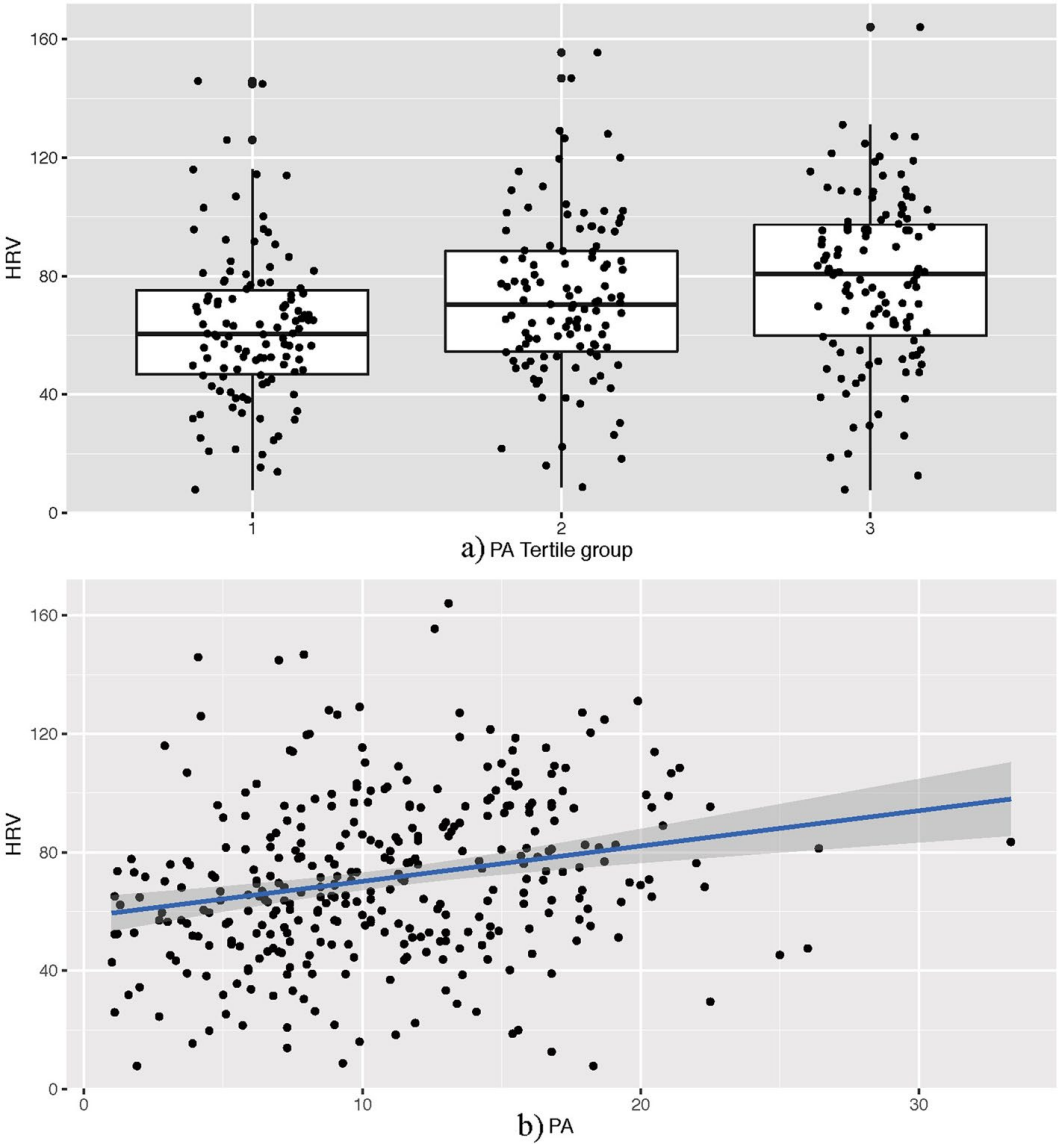
Box and scatter plots of the association between PA and HRV. HRV, heart rate variability; PA, physical activity

The distribution of HRV among groups with different PA levels is displayed using the box plot (Fig. 3a), with HRV increasing from PA tertile 1 group (HRV, median = 60.4 ms, interquartile range [IQR]: 46.5–75.8 ms) to PA tertile 2 group (HRV, median = 70.4 ms, IQR: 54.3–88.5 ms) to PA tertile 3 group (HRV, median = 80.7 ms, IQR: 59.2–97.8 ms). A positive linear association between PA and HRV is demonstrated using the scatter plot (Fig. 3b).

A simple linear regression model was used to further assess the linear association between PA and HRV, and the β of HRV was 1.193 (95%CI: 0.657–1.729, *P* < 0.001). After adjusting for potential confounding variables in the multiple linear regression analysis, the β of HRV was 0.842 (95%CI: 0.261–1.425, *P* = 0.005), indicating that HRV was increased by 0.842 ms for each additional 1% of PA at baseline.

### Cutoff values of HRV and PA for predicting long-term outcomes

**Fig. 4:**
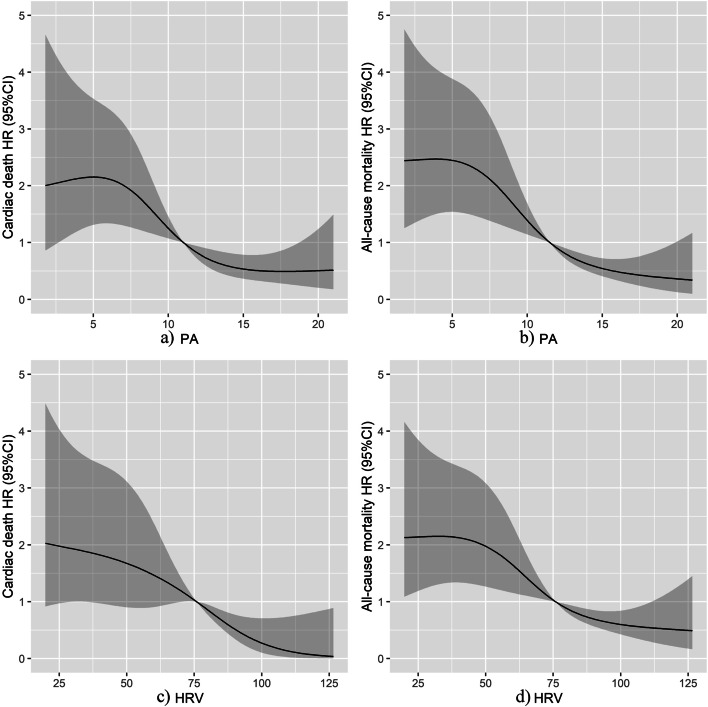
Relationships between HRV/PA and HRs for cardiac death and all-cause mortality. 3a) smoothing curve fitting of PA in predicting the risks of cardiac death; 3b) smoothing curve fitting of PA in predicting the risks of all-cause mortality; 3c) smoothing curve fitting of HRV in predicting the risks of cardiac death; 3d) smoothing curve fitting of HRV in predicting all-cause mortality. HR, hazard ratio; HRV, heart rate variability; PA, physical activity

The associations between PA or HRV and HRs for cardiac death and all-cause mortality are demonstrated using the smooth curve fitting (Fig. 4). The risks of cardiac death and all-cause mortality declined rapidly before 75.9 ms of HRV and 11.0% of PA and then started to decrease slowly thereafter. Thus, 75.9 ms of HRV and 11.0% of PA were obtained as the cut-off values.

### Predictive values of PA for long-term outcomes

Univariable and multivariable Cox regression analyses revealed that increased PA protected against the risks of cardiac death and all-cause mortality (Table 2). In multivariable Cox regression model 1, a high level of PA (≥11.0%) was associated with reduced risks of cardiac death (HR = 0.239; 95% CI: 0.124–0.460, *P* < 0.001) and all-cause mortality (HR = 0.277; 95% CI: 0.163–0.469, P < 0.001) after adjusting for age at implantation, sex, BMI, LVEF, LVEDD, ICD or CRT-D implantation, NYHA Class, hypertension, DM, stroke, DCM, ICM, MI, PCI, pre-implant syncope, prior AF, use of ACEIs/ARBs, use of diuretics, and use of aldosterone antagonists. When adjusting for the above-mentioned variables and additional HRV, the predictive values of PA remained significant for cardiac death (HR = 0.897; 95% CI: 0.844–0.954, *P* = 0.001) and all-cause mortality (HR = 0.888; 95% CI: 0.844–0.933, P < 0.001) in multivariable model 2, indicating that each 1% increase in PA could result in a 10.3 and 11.2% reduction in the risks of cardiac death and all-cause mortality, respectively.

**Table 2:** Predictive values of PA for long-term mortality

	Univariable Cox regression model		Multivariable Cox regression model 1		Multivariable Cox regression model 2	
	HR (95% CI)	P value	HR (95% CI)	P value	HR (95% CI)	P value
**Primary endpoint: cardiac death**						
Per 1%/increase^a^	0.904 (0.858–0.952)	*P* < 0.001	0.887 (0.834–0.944)	*P* < 0.001	0.897 (0.844–0.954)	P = 0.001
≥11.0% group vs < 11.0% group	0.280 (0.154–0.509)	*P* < 0.001	0.239 (0.124–0.460)	P < 0.001	0.273 (0.142–0.526)	P < 0.001
**Secondary endpoint: all-cause mortality**						
Per 1%/increase^a^	0.886 (0.848–0.926)	P < 0.001	0.880 (0.836–0.926)	*P* < 0.001	0.888 (0.844–0.933)	*P* < 0.001
≥11.0% group vs < 11.0% group	0.276 (0.170–0.449)	P < 0.001	0.277 (0.163–0.469)	P < 0.001	0.299 (0.177–0.505)	P < 0.001

Multivariable Cox regression model 1 was adjusted for age at implantation, sex, BMI, LVEF, LVEDD, ICD or CRT-D implantation, NYHA Class, hypertension, DM, stroke, DCM, ICM, MI, PCI, pre-implant syncope, prior AF, use of ACEIs/ARBs, use of diuretics, and use of aldosterone antagonists. Multivariate Cox regression model 2 was adjusted for the above-mentioned confounders and additional HRV

*ACEIs/ARBs* angiotensin-converting enzyme inhibitors or angiotensin receptor blockers, *AF* atrial fibrillation, *BMI* body mass index, *CI* confidence interval, *CRT-D* cardiac resynchronisation therapy defibrillator, *DM* diabetic mellitus, *HRV* heart rate variability, *HR* hazard ratio, *ICD* implantable cardioverter defibrillator, *ICM* ischaemic cardiomyopathy, *LVEF* left ventricular ejection fraction, *LVEDD* left ventricular end-diastolic dimension, *MI* myocardial infarction, *NYHA* New York Heart Association, *PA* physical activity, *PCI* percutaneous coronary intervention

^a^ each additional 1% increase in PA

### Predictive values of HRV for long-term outcomes

In 342 ICD/CRT-D recipients, univariable and multivariable Cox regression analyses showed that HRV was inversely associated with the incidence rates of cardiac death and all-cause mortality (Table 3). Multivariable Cox regression analyses revealed that a high level of HRV (≥75.9 ms) was associated with reduced risks of cardiac death (HR = 0.197; 95% CI: 0.090–0.428, *P* < 0.001) and all-cause mortality (HR = 0.351; 95% CI: 0.205–0.600, P < 0.001) when adjusted for age at implantation, sex, BMI, LVEF, LVEDD, ICD or CRT-D implantation, NYHA Class, hypertension, DM, stroke, DCM, ICM, MI, PCI, pre-implant syncope, prior AF, use of ACEIs/ARBs, use of diuretics, and use of aldosterone antagonists. After adjusting for the above-mentioned variables and additional PA in multivariable model 2, the predictive values of HRV remained significant for cardiac death (HR = 0.979; 95% CI: 0.968–0.991, P < 0.001) and all-cause mortality (HR = 0.986; 95% CI: 0.978–0.995, *P* = 0.003), indicating that each 1 ms increase in HRV could result in a 2.1 and 1.4% reduction in the risks of cardiac death and all-cause mortality, respectively.

**Table 3:** Predictive values of HRV for long-term mortality

	Univariable Cox regression model		Multivariable Cox regression model 1		Multivariable Cox regression model 2	
	HR (95% CI)	P value	HR (95% CI)	***P*** value	HR (95% CI)	P value
**Primary endpoint: cardiac death**						
Per 1 ms/increase^a^	0.978 (0.968–0.987)	P < 0.001	0.978 (0.967–0.989)	P < 0.001	0.979 (0.968–0.991)	P < 0.001
≥75.9 ms group vs < 75.9 ms group	0.169 (0.080–0.355)	P < 0.001	0.197 (0.090–0.428)	P < 0.001	0.224 (0.103–0.489)	P < 0.001
**Secondary endpoint: all-cause mortality**						
Per 1 ms/increase^a^	0.983 (0.976–0.991)	P < 0.001	0.984 (0.976–0.993)	P < 0.001	0.986 (0.978–0.995)	P = 0.003
≥75.9 ms group vs < 75.9 ms group	0.291 (0.176–0.482)	P < 0.001	0.351 (0.205–0.600)	P < 0.001	0.394 (0.231–0.674)	P = 0.001

Multivariable Cox regression model 1 was adjusted for age at implantation, sex, BMI, LVEF, LVEDD, ICD or CRT-D implantation, NYHA Class, hypertension, DM, stroke, DCM, ICM, MI, PCI, pre-implant syncope, prior AF, use of ACEIs/ARBs, use of diuretics, and use of aldosterone antagonists. Multivariate Cox regression model 2 was adjusted for the above-mentioned confounders and additional PA

*ACEIs/ARBs* angiotensin-converting enzyme inhibitors or angiotensin receptor blockers, *AF* atrial fibrillation, *BMI* body mass index, *CI* confidence interval, *CRT-D* cardiac resynchronisation therapy defibrillator, *DM* diabetic mellitus, *HRV* heart rate variability, *HR* hazard ratio, *ICD* implantable cardioverter defibrillator, *ICM* ischaemic cardiomyopathy, *LVEF* left ventricular ejection fraction, *LVEDD* left ventricular end-diastolic dimension, *MI* myocardial infarction, *NYHA* New York Heart Association, *PA* physical activity, *PCI* percutaneous coronary intervention

^a^ each additional 1 ms increase in HRV

### Mediation analysis

Figure 1 summarises the results of causal mediation analysis. In Path a, PA (independent variable) had a positive linear relationship with HRV (mediator) in a multiple linear regression analysis. In Path b, when HRV was not included in the multivariable Cox regression model, PA (independent variable) was an independent predictor for long-term cardiac death and all-cause mortality (outcome variables). In Path c, both PA (independent variable) and HRV (mediator) remained significant to predict the outcome variables in the multivariable Cox regression model. Thus, this mediation analysis confirmed the partial mediation function of HRV in the association between high levels of PA and increased risks of cardiac death and all-cause mortality.

**Fig. 5:**
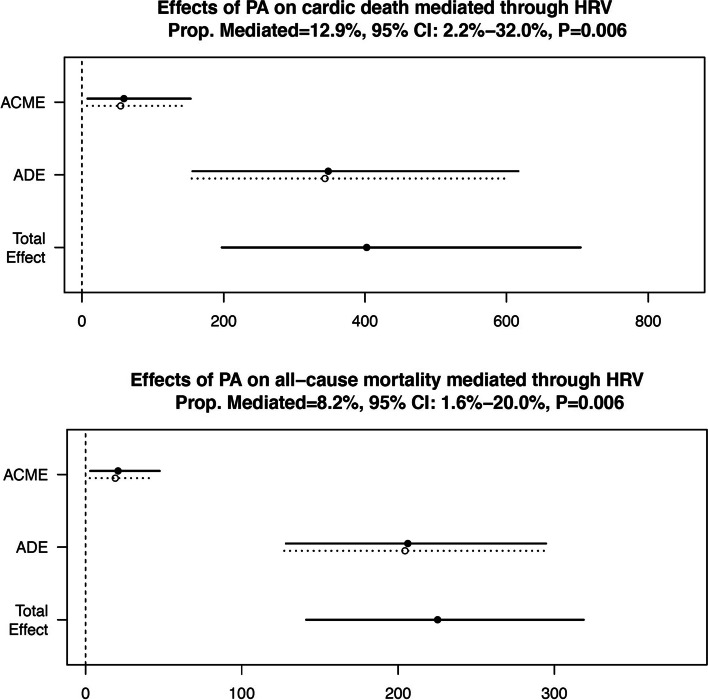
Causal mediation analysis results. ACME stands for average causal mediation effects of PA on the long-term mortality mediated through HRV; ADE stands for average direct effects of PA on the long-term mortality; Total effect stands for the total effects (direct and indirect) of PA on the long-term mortality; Prop. Mediated describes the proportion of the indirect/mediation effect of PA on the long-term mortality that goes through the mediator. ACME, average causal mediation effect; ADE, average direct effect; CI, confidence interval; HRV, heart rate variability; PA, physical activity; Prop. Mediated: mediation proportion

Figure 5 shows that for cardiac death, the mediation effect was statistically significant (ACME = 56.7, 95%CI: 7.4–148.7, *P* = 0.006; total effect = 402.3, 95%CI: 197.3–705.1, *P* < 0.001), and 12.9% (prop. Mediated = 12.9, 95%CI: 2.2–32.0%, P = 0.006) of the mediation effect of PA on the risks of cardiac death was mediated through HRV. For all-cause mortality, the mediation effect remained significant (ACME = 20.0, 95%CI: 2.9–45.6, P = 0.006; total effect = 225.2, 95%CI: 141.2–318.7, P < 0.001), and 8.2% (prop. Mediated = 8.2, 95%CI: 1.6–20.0%, P = 0.006) of the mediation effect of PA on the risks of all-cause mortality was mediated through HRV.

## Discussion

In this retrospective analysis, data regarding PA and HRV obtained from a continuous remote home monitoring system were included, and causal mediation analysis was established to explore the possible mediation function of HRV in the association between PA and long-term mortality in patients with a high risk of sudden cardiac death (SCD). The main findings were: 1) HRV was positively associated with PA, 2) both PA and HRV were independent protective predictors for cardiac death and all-cause mortality, 3) the causal mediation analysis confirmed partial mediation effects of PA on the risks of cardiac death and all-cause mortality among 342 ICD/CRT-D recipients; 12.9 and 8.2% of the mediation effects of PA on the risks of cardiac death and all-cause mortality, respectively, were mediated through HRV. These findings support that PA might contribute to cardiovascular benefits partly via enhanced cardiac autonomic function.

The autonomic nervous system plays a crucial role in the clinical course of CVDs through modulating arrhythmias, heart rate, blood pressure, breathing, digestion, etc. [14–17]. HRV is the fluctuation in the intervals between heartbeats, which can be acquired using the time-domain or frequency-domain measurement from ECG recordings [14, 15]. High levels of HRV can reflect better modulation between sympathetic and parasympathetic activities [14, 15]. Some studies conducted cross-sectional analyses to investigate the association between cardiac autonomic function and PA in the general population at different ages [12, 13, 28, 29]. Soares-Miranda et al. obtained HRV indices from a 24-h Holter in 985 older adults in the Cardiovascular Health Study [28]. Data regarding PA, which was categorised as leisure-time activity, walking distance, and walking pace, were obtained using the modified Minnesota PA questionnaire. Greater PA demonstrated an association with more favourable HRV indices in older adults. In addition, the Finnish MOPD study and a British study of civil servants focused on 3629 adolescent men and 3328 middle-aged (35–55 years) people, respectively [12, 29]. HRV was derived from 5-min ECG recordings. Moderate and vigorous PA were observed associated with higher HRV indices in both studies. In the above-mentioned studies, HRV values was acquired from traditional short-term ECG recordings, or PA was obtained from self-assessment questionnaires [12, 13, 28, 29]. Differently, Zhao S et al. obtained the objective accelerometer-measured PA and time-dominant HRV values from continuous ECG recordings in ICD/CRT-D recipients, and confirmed a significant correlation (r = 0.601, *P* < 0.001) between PA and HRV although the sample size was small [11]. In the present study, both HRV and PA values were derived from continuous and quantitative monitoring data and their average daily values were measured during the first 30–60 days after ICD/CRT-D implantation for each patient, as recommended by published evidence [11, 20]. Box and scatter plot analyses, and multiple linear regression analysis were conducted to further investigate the association between PA and HRV. The results showed PA had a robust positive linear relationship with HRV, which was consistent with previous findings. Therefore, the present study provided more evidence to support that regular physical exercise was related to enhanced cardiac autonomic regulation. Although the cellular and molecular interpretations of the positive linear relationship between cardiac autonomic and PA are complex, PA may modulate cardiac autonomic function by improving cardiomyocyte contractile capacity and cardiac electrical stability [30, 31].

Previous studies have clarified the predictive values of HRV or PA for long-term outcomes in patients with different CVDs [3–7, 16, 17, 20, 32–34]. Whether PA was self-reported or accelerometer-derived, low levels of PA were associated with increased incidences of hospitalisations for HF, cardiovascular adverse events, cardiovascular death, and all-cause mortality [3–7, 20]. In previous studies on different populations, HRV values were derived from 10-s to 24-h ECG recordings [16, 17]. A low level of HRV, indicative of cardiac autonomic dysfunction, was identified as an independent risk factor for ventricular arrhythmia in patients who had experienced acute MI and in those who were at a high risk of SCD [16, 17, 32]. Other studies have reported similar results for cardiac death and all-cause mortality in older adults and patients at a high risk of type 2 DM [33, 34]. However, the predictive values of PA and HRV for long-term mortality were not analysed in the same study population. Moreover, the mediation function of improved HRV in the relationship between regular PA and satisfying clinical outcomes was not explored. Schwartz et al. observed that 6 weeks of treadmill training reduced the incidence of ventricular fibrillation during acute myocardial ischaemia by 100%, while HRV increased by 74% derived from 25-min ECG recordings [35]. It implied that the underlying mechanism of 100% survival rate might be that exercise training improved cardiac autonomic modulation [35]. In the present study, the association between PA and HRV, and their effects on cardiac death and all-cause mortality were simultaneously analysed in ICD/CRT-D recipients. Additionally, we conducted a causal mediation analysis to explore the possible mediation function of cardiac autonomic function in the association between PA and long-term cardiac death and all-cause mortality.

In the multivariable Cox regression analysis, both HRV and PA were found to be independent predictors for cardiac death and all-cause mortality, regardless of various baseline characteristics. Every additional 1% increase in PA could result in a 10.3 and 11.2% reduction in risks of cardiac death and all-cause mortality, respectively; every additional 1 ms increase in HRV could result in a 2.1 and 1.4% reduction in risks of cardiac death and all-cause mortality, respectively. The partial mediation function was confirmed using the causal mediation analysis following the Baron and Kenny’s procedure [24]. It was further estimated that the proportions of indirect effects of PA on the risks of cardiac death and all-cause mortality mediated through cardiac autonomic nervous modulation were 12.9 and 8.2%, respectively. This finding revealed that the underlying mechanisms were multifactorial and complex, and enhanced cardiac autonomic regulation might be one of the most important mechanisms by which regular PA contributed to cardiovascular benefits. Cardiac autonomic regulation should be emphasised in the management of patients after ICD/CRT-D implantation. More effective exercise regimens to enhance the cardiac autonomic function should be developed in the clinical practice. In contrast, the direct effects of PA, which probably include improved cardiac output, enhanced cardiorespiratory fitness, reduced systemic vascular resistance, and regulation on cardiac remodelling, etc., cannot be ignored [36].

### Limitations

Two potential limitations were considered in the present study. First, in the present study, only observational data regarding PA and HRV from ICD/CRT-D recipients were retrospectively analysed. Interventional studies with a larger sample size are required to further explore the mediation function of HRV in the association between high levels of PA and long-term mortality in different populations. Second, selection bias can occur, and caution should be exercised while generalizing the results to populations other than ICD/CRT-D recipients. Third, HRV indices were acquired in a time-dominant form using SDNN algorithm. SDNN algorithm can allow for precise interpretations of cardiac autonomic function and no gold standards for HRV measurement exist [15]. However, it would be better if both frequency-dominant and time-dominant HRV indices could be evaluated.

## Conclusions

Causal mediation analysis revealed partial mediation function of HRV in the association between PA and long-term mortality in ICD/CRT-D recipients. About 12.9 and 8.2% of the mediation effects of PA on the risks of cardiac death and all-cause mortality, respectively, were mediated through HRV. These findings indicated that enhanced cardiac autonomic function might be one of the underlying mechanisms by which regular PA contributed cardiovascular benefits.

## Data Availability

The datasets generated and analysed during the current study are not publicly available due to the Fuwai Hospital regulations but are available from the corresponding author on reasonable request.

## Abbreviations

ACEI: angiotensin-converting enzyme inhibitor
ACME: average causal mediation effect
ADE: average direct effect
AF: atrial fibrillation
ARB: angiotensin receptor blocker
BMI: body mass index
CABG: coronary artery bypass grafting
CCB: calcium channel blocker
CI: confidence interval
CRT-D: cardiac resynchronisation therapy defibrillator
CVD: cardiovascular disease
DCM: dilated cardiomyopathy
DM: diabetes mellitus
ECG: electrocardiography
HCM: hypertrophic cardiomyopathy
HF: heart failure
HR: hazard ratio
HRV: heart rate variability
ICD: implantable cardioverter defibrillator
ICM: ischaemic cardiomyopathy
LVEDD: left ventricular end-diastolic dimension
LVEF: left ventricular ejection fraction
LQTS: long QT syndrome
MI: myocardial infarction
NYHA: New York Heart Association
PA: physical activity
PCI: percutaneous coronary intervention
Prop. Mediated: mediation proportion
SCD: sudden cardiac death
SD: standard deviation
SUMMIT: Study of Home Monitoring System Safety and Efficacy in Cardiac Implantable Electronic Device-implanted Patients
